# Effect of parathyroid hormone-related protein on odontogenic differentiation in human dental pulp cells

**DOI:** 10.1186/s12903-020-01085-8

**Published:** 2020-04-10

**Authors:** Mi-Ra Kim, Sung-Hyeon Choi, Bin-Na Lee, Kyung-San Min, Yun-Chan Hwang

**Affiliations:** 1 Department of Conservative Dentistry, School of Dentistry, Dental Science Research Institute, Chonnam National University, Youngbong-ro 77, Buk-gu, Gwangju, 61186 South Korea; 2 Department of Conservative Dentistry, School of Dentistry, Jeonbuk National University, 567 Baekje-daero, Deokjin-gu, Jeonju, 54896 South Korea

**Keywords:** PTHrP, Odontogenic differentiation, Mineralization

## Abstract

**Background:**

Parathyroid hormone-related protein (PTHrP) plays an important role in many physiological processes, including bone regeneration. The function of PTHrP is similar to PTH. It promotes osteogenic differentiation in MC3T3-E1 cells. The aim of this study was to investigate whether PTHrP might have odontogenic differentiation ability in human dental pulp cells (hDPCs).

**Methods:**

The viability of hDPCs after stimulation with PTHrP was measured. Real-time polymerase chain reaction and Western blot analysis were performed to evaluate the expression levels of odontogenic markers and activation of protein kinase B (PKB/AKT), extracellular signal-regulated kinase (ERK), c-Jun N-terminal kinase (JNK), and p38 mitogen-activated protein kinase (MAPK). To evaluate mineralized nodule formation, alkaline phosphatase (ALP) staining and alizarin red S staining were performed.

**Results:**

PTHrP promoted odontogenic differentiation as evidenced by the formation of mineralized nodules, the induction of ALP activity, and the upregulation of odontogenic markers (dentin sialophosphoprotein and dentin matrix protein-1). The phosphorylation of AKT, ERK, JNK, and p38 was increased by PTHrP. However, an AKT inhibitor (LY294002), an ERK inhibitor (U0126), a JNK inhibitor (SP600125), and a p38 inhibitor (SB203580) inhibited the increase of mineralization induced by PTHrP.

**Conclusion:**

The present study revealed that PTHrP could promote odontogenic differentiation and mineralization through activating the AKT, ERK, JNK, and p38 signaling pathways. These results provide novel insights into the odontogenic action of PTHrP.

## Background

Dentin is a major component of teeth. It shows strong regenerative potential [1]. When infected dentin is removed, the pulp may be exposed. Regeneration therapy, such as direct pulp capping, can keep pulp viable and form a physical barrier. It could function as a biological seal between dental material and pulp tissue [2, 3]. Successful pulp capping is very important and is affected by several factors. Growth factors play a key role in cell survival, proliferation, and differentiation for the successful regeneration of pulp-dentin complexes [3, 4].

Dental pulp stem cells are clonogenic cells capable of both self-renewal and multiple lines of differentiation [5]. Dental pulp cells can differentiate into odontoblasts that act as precursor cells important for dentin formation [6, 7]. Several studies have shown that biologically active components such as osteostatin can enhance the osteogenic differentiation and mineralization of osteoblastic cells that are responsible for new bone formation [8]. Similar to bone formation, osteostatin can lead to reparative dentin formation by inducing osteoblast-like human dental pulp stem cells (hDPCs) [9].

Parathyroid hormone-related protein (PTHrP) can induce bone formation. A previous study reported that the osteogenic differentiation of MC3T3-E1 cells could be promoted by the bone-forming ability of PTHrP at various concentrations [10]. PTHrP is a major contributor to hypercalcemia. It is similar to PTH structurally and functionally [11, 12]. It influences chondrocytic and osteogenic cell biology and plays an important role in bone remodeling, the regulation of fetal blood calcium, and many physiologic processes [13–15]. PTHrP can increase the expression levels of COL2A1 and Sox9, known to be involved in chondrogenic differentiation in chondrogenic medium in mesenchymal stem cells. It can significantly enhance cartilage formation and upregulate chondrocyte proliferation through cyclin-dependent kinase inhibition [16–18].

Previous studies have demonstrated that PTHrP 1–141 and PTHrP 1–86 possess anabolic action, indicating that osteogenic differentiation could be promoted in MC3T3-E1 cells by assessing the osteogenic ability of PTHrP at varying concentrations [12, 19]. In contrast, PTHrP homozygous mutants caused abnormalities in endochondral bone growth with short ribs and malformed long bones [20, 21]. Several studies have shown that PTHrP activated signaling pathways, leading to the activation of several transcription factors that play important roles in signal transduction in osteoblasts [22–24].

The odontogenic potential of PTHrP has not yet been reported. Therefore, the aim of this study was to investigate the underlying signaling mechanisms of PTHrP-mediated odontogenic differentiation.

## Methods

### Cell isolation and culture of hDPCs

This study was approved by the Institutional Review Board of Chonnam National University Dental Hospital, Gwangju, Korea (IRB No. CNUDH-2016-009). Written informed consent was obtained from each patient included in this study. Extracted human third molars with pulp tissues were obtained from the Department of Oral Maxillofacial Surgery, Chonnam National University Dental Hospital. Tooth samples were removed aseptically, rinsed with Dulbecco’s phosphate-buffered saline solution (DPBS, Welgene, Daegu, South Korea), and placed in 60 mm dishes. The cells were cultured in growth media (GM) consisting of α-minimum essential medium (α-MEM, Gibco Invitrogen, Grand Island, NY, USA) supplemented with 10% fetal bovine serum (FBS, Gibco Invitrogen) and 1% antibiotics (100 U/mL penicillin and 100 mg/mL streptomycin, Gibco Invitrogen) in a humidified atmosphere of 5% CO_2_ at 37 °C. For mineralization experiments, the cells were cultured in odontogenic induction medium (OM) containing 50 mg/mL ascorbic acid (Sigma-Aldrich, St. Louis, MO, USA) and 10 mmol/L β-glycerophosphate (Santa Cruz Biotechnology, Inc., Dallas, TX, USA). When the cells reached confluence, subcultures were performed and cells with passage numbers 3 to 4 were used for this study and rinsed with DPBS. The cells were detached with an appropriate quantity of trypsin solution (Gibco Invitrogen) for three minutes in a CO_2_ incubator in a humidified atmosphere of 5% CO_2_ at 37 °C. The cell suspension was transferred to a tube and gently centrifuged at 800 RPM for five minutes. After removing the supernatant, the cell pellet was gently resuspended in GM consisting of 10% α-MEM. The density of the viable cells was determined by counting in a hemocytometer and the cells were seeded in wells or dishes in the following experiments.

### PTHrP treatment

Recombinant human PTHrP (Sigma-Aldrich) was directly added at 1 nM or 10 nM to the OM. The cells were treated with PTHrP the next day after seeding and incubated with PTHrP for 3, 5, 7, or 14 days. The medium was changed with fresh medium containing PTHrP every two days.

### Cytotoxicity test

HDPCs were seeded at a density of 1 × 10^4^cells per well in 96-well culture plates. After 24 h of culture, the cells were treated with PTHrP at different concentrations (1, 10, and 100 nM) for 24 h. After 24 h of culture, cell viability was analyzed by the WST-1 assay using an EZ-Cytox Enhanced Cell Viability Assay Kit (Daeil Lab Service, Seoul, Korea). Briefly, 10 μL of EZ-Cytox reagent was added to each well in the 96-well plate and incubated at 37 °C for four hours. The absorbance was measured at a wavelength of 420 nm using a spectrophotometer (Thermo Scientific Multiskan GO, Waltham, MA, USA).

### RNA extraction and quantitative real-time polymerase chain reaction assay

HDPCs were seeded at a density of 2 × 10^5^ cells per well in 6-well culture plates with GM. After 24 h of culture, the cells were treated in OM with or without 1 and 10 nM PTHrP for three and five days. Total RNA was then extracted from the cells using Trizol reagent (Gibco Invitrogen) according to the manufacturer’s recommended protocol. Complementary DNA (cDNA) was synthesized using a random primer (Promega Biotech, Piscataway, NJ, USA) and an AccessQuick™ real-time polymerase chain reaction (RT-PCR) system (Promega, Madison, WI, USA). Quantitative RT-PCR was performed using a QuantiTect SYBR Green PCR Kit (Qiagen, Valencia, CA, USA) on a 72-well Rotor-Gene 6000 (Corbett Research, Sydney, Australia). The primer sequences used for PCR are listed in Table 1. The relative gene expression levels were analyzed using the 2^-∆∆Ct^ method. To examine the effect of PTHrP, the same assay described above was performed for cells cultured in OM with or without PTHrP at 1 or 10 nM.

**Table 1:** List of primers used for real-time PCR

Gene	Sequences (5′-3′)
DSPP	Forward: GGG AAT ATT GAG GGC TGG AA
	Reverse: TCA TTG TGA CCT GCA TCG CC
DMP-1	Forward: TGG TCC CAG CAG TGA GTC CA
	Reverse: TGT GTG CGA GCT GTC CTC CT
β-actin	Forward: CTC CTT AAT GTC ACG CAC GAT
	Reverse: CCT TGT AGC CAG GCC CAT TG

### Western blot analysis

HDPCs were seeded at a density of 3 × 10^5^ cells per dish on 60 mm cell culture dishes and cultured in OM with or without PTHrP at 1 or 10 nM. The medium was replaced with fresh medium every two days. The PTHrP-induced odontogenic protein expression and activation of protein kinase B (PKB/AKT), extracellular signal-regulated kinase (ERK), c-Jun N-terminal kinases (JNK), and p38 mitogen-activated protein kinase (MAPK) were analyzed by Western blots. HDPCs were treated with or without PTHrP at 1 or 10 nM. The cells were washed twice with PBS and extracted with cell lysis buffer (Cell Signaling Technology, Beverly, MA, USA). Then cell lysates were then centrifuged at 13,000 rpm for 10 min. The supernatants were collected and protein concentrations were determined using a Lowry Protein Assay Reagent Kit (Bio-Rad Laboratories, Hercules, CA, USA). The proteins were subjected to 10% sodium dodecyl sulfate–polyacrylamide gel electrophoresis at 80 V for two hours and transferred to polyvinylidene difluoride membranes at 10 V overnight. After blocking with 5% non-fat dried skim milk in PBS containing 0.1% Tween 20 (PBST) at room temperature for one hour, the membranes were incubated with anti–dentin sialophosphoprotein (DSPP) (1:2000; Thermo Fisher Scientific), anti–dentin matrix protein (DMP)-1 (1:2000; Abcam, Cambridge, UK), anti-ERK (1:2000; Cell Signaling Technology), and anti-phospho-ERK (1:2000; Cell Signaling Technology) at 4 °C overnight. After washing three times with PBST, the membranes were then incubated with horseradish peroxidase (HRP)-conjugated anti-rabbit IgG secondary antibodies (1:10,000 Sigma-Aldrich) at room temperature for one hour. After washing with PBS five times, chemiluminescent HRP substrate (Millipore, Billerica, MA, USA) was used to visualize the protein signals with a Chemiluminescence Imaging System (Ez-capture; Atto, Tokyo, Japan).

### Alkaline phosphatase staining assay

HDPCs were seeded at a density of 2 × 10^4^cells per well in 24-well culture plates and cultured in GM and OM with or without PTHrP at 1 or 10 nM for seven days. Fresh GM and OM were replaced every two days. After seven days of exposure, the medium was removed. The cells were washed with PBS and fixed in 70% ethanol for one hour, followed by rinsing with distilled water three times. The fixed cells were treated with 300 μL alkaline phosphatase (ALP) staining reagent (1-step NBT/BCIP solution; Thermo Fisher Scientific) per well. After removing the staining reagent, a photograph was taken using an Officejet Pro L7580 scanner (Hewlett-Packard, Palo Alto, CA, USA). To quantitatively evaluate the staining results, the stain was treated with 10% cetylpyridinium chloride (pH = 7.0) for 30 min at room temperature, followed by absorbance measurement at a wavelength of 562 nm using a spectrophotometer (Thermo Scientific Multiskan GO).

### Alizarin red staining assay

HDPCs were seeded at a density of 2 × 10^4^cells per well in 24-well cell culture plates and cultured in GM and OM with or without PTHrP at 1 or 10 nM for 14 days. After 14 days, the cells were fixed in 70% ethanol and stained with 2% alizarin red S staining reagent (LIFELINE Cell Tech, Frederick, MD, USA). The results were recorded using an Officejet Pro L7580 scanner. For quantitative analysis, 10% cetylpyridinium chloride (pH = 7.0) was added to each sample and the absorbance value was measured at a wavelength of 540 nm using a spectrophotometer (Thermo Scientific Multiskan GO).

### Statistical analysis

Three independent experiments were performed. The results are expressed as means ± standard deviation of the means. Statistical significance was determined by one-way analysis of variance. All statistical analyses were performed using SPSS 23.0 software program (SPSS, Chicago, IL, USA). Differences were considered statistically significant at *P* < 0.05.

## Results

### Effects of PTHrP on cell viability of hDPCs

**Fig. 1:**
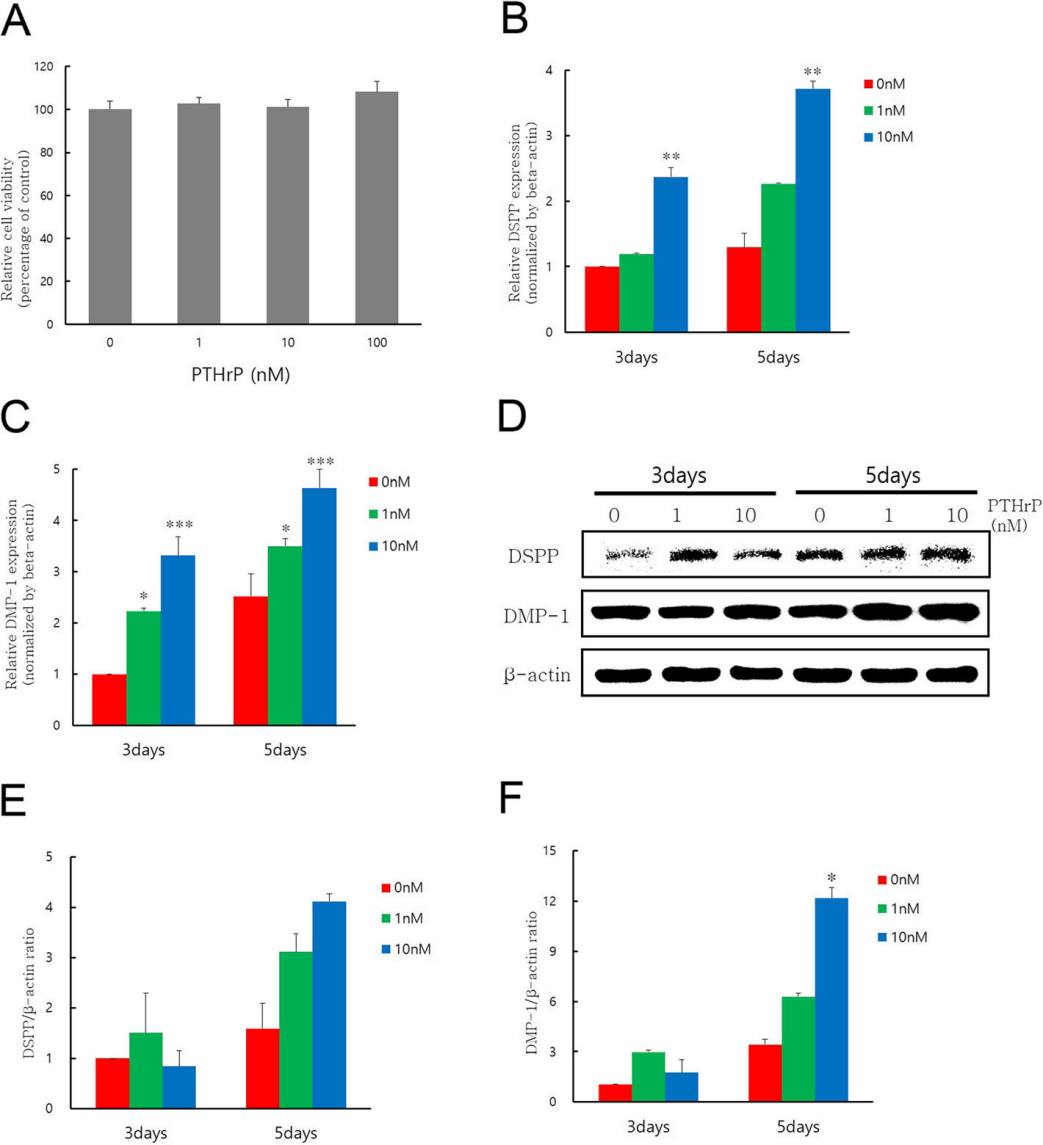
**a** Results of water-soluble tetrazolium salt-1 assay for assessing the viabilities of hDPCs treated with PTHrP at different concentrations (1, 10, and 100 nM). The effects of PTHrP at 1 and 10 nM on mRNA expression levels of odontogenic markers were determined by quantitative reverse transcription-polymerase chain reaction. The expression levels of **b** DSPP and **c** DMP-1 in hDPCs stimulated with PTHrP at 1 or 10 nM over different time periods were determined. **d** DSPP and DMP-1 protein levels were increased after treatment with 1 nM or 10 nM of PTHrP for three and five days compared to those in the control without PTHrP treatment. (E-F) Graphic representation of DMP-1 and DSPP protein levels. These graphs were normalized to data from cells not treated with PTHrP. ^*^Statistically significant difference compared to the no PTHrP group (*P* < 0.05), ^**^Statistically significant difference compared to the no PTHrP group (*P* < 0.01). ^***^Statistically significant difference compared to the no PTHrP group (*P* < 0.001)

A WST-1 assay was performed to evaluate the cell viability of hDPCs treated with PTHrP at 1, 10, and 100 nM. The cell viability was not significantly different between the untreated group and the groups treated with different concentrations of PTHrP (Fig. 1a).

### Effects of PTHrP on Odontogenic differentiation and mineralization in hDPCs

Quantitative RT-PCR analyses were performed to investigate the effect of PTHrP on odontogenic differentiation in hDPCs. The DSPP and DMP-1 mRNA expression levels were gradually increased with increasing concentrations of PTHrP three and five days after treatment. DSPP mRNA levels were significantly increased in the group treated with 10 nM PTHrP compared to the group not treated with PTHrP (^**^P < 0.01) (Fig. 1b). DMP-1 mRNA levels were also significantly increased after treatment with PTHrP at concentration of 1 or 10 nM compared to the group not treated with PTHrP (^*^P < .05 and ^***^P < .001) (Fig. 1c).

Western blot analyses were performed to investigate the effect of PTHrP on the odontogenic differentiation in hDPCs. As shown in Fig. 1d, the protein levels of DSPP in the PTHrP-treated cells were higher than those in the no PTHrP group on days 3 and 5, although the difference was not statistically significant. The protein levels of DMP-1 were increased significantly in the group treated with 10 nM PTHrP on day 5 and slightly increased on day 3.

**Fig. 2:**
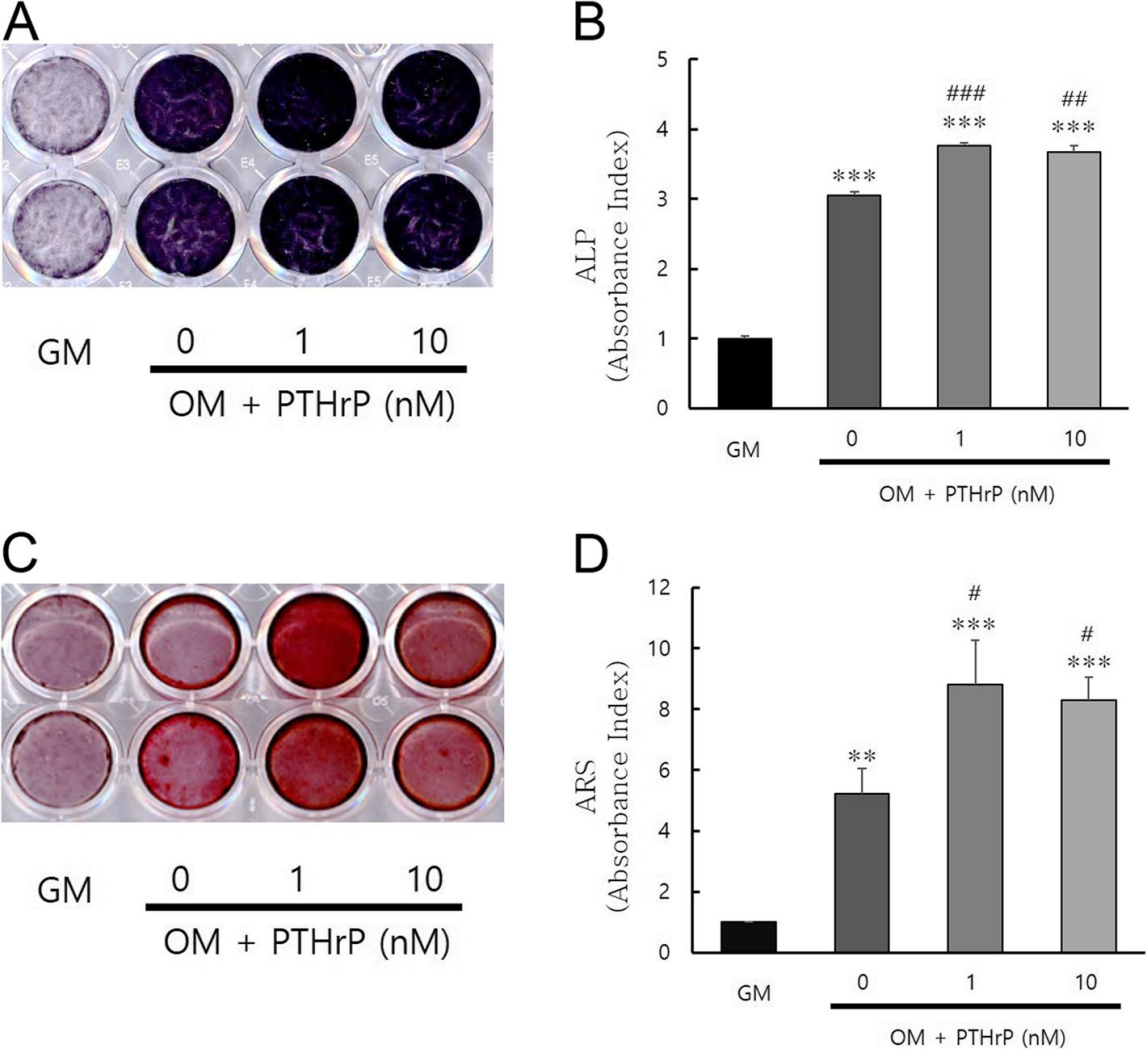
Effect of PTHrP on mineralization in hDPCs. (**a**) Cells cultured with or without PTHrP at 1 or 10 nM for seven days. ALP activity was evaluated by ALP staining. (**b**) Quantification of ALP staining. ALP staining was increased after PTHrP treatment for seven days compared to the GM control without PTHrP treatment. (**c**) Cells cultured with or without PTHrP at 1 or 10 nM for 14 days. Calcium nodule deposition was evaluated by Alizarin red S staining. (**d**) Quantification of Alizarin red S staining. Mineralized nodule formation was significantly increased after PTHrP treatment compared to the GM and the OM groups without PTHrP treatment. The graphs were normalized to GM (^*^P < 0.05, ^**^P < 0.01, and ^***^P < 0.001 compared to GM group; ^#^*P* < 0.05, ^##^*P* < 0.01, and ^###^*P* < 0.001 compared to the OM group without PTHrP treatment)

To verify the mineralization effect of PTHrP on hDPSCs, ALP staining and Alizarin red staining with or without 1 or 10 nM PTHrP treatment were performed. After seven days, ALP staining was significantly (^***^P < 0.001) increased in all groups compared to that in the GM group. There was also a significant difference in ALP staining in the 1 nM or 10 nM PTHrP groups compared to the no PTHrP group (^##^*P* < 0.01 and ^###^*P* < 0.001). After 14 days, treatment with 1 and 10 nM PTHrP significantly enhanced calcium nodule deposition based on Alizarin red staining compared to the GM or no PTHrP groups (^#^*P* < 0.05, ^**^P < 0.01 and ^***^P < 0.001) (Fig. 2).

### Effects of PTHrP on AKT, ERK, JNK, and p38 signaling pathways

**Fig. 3:**
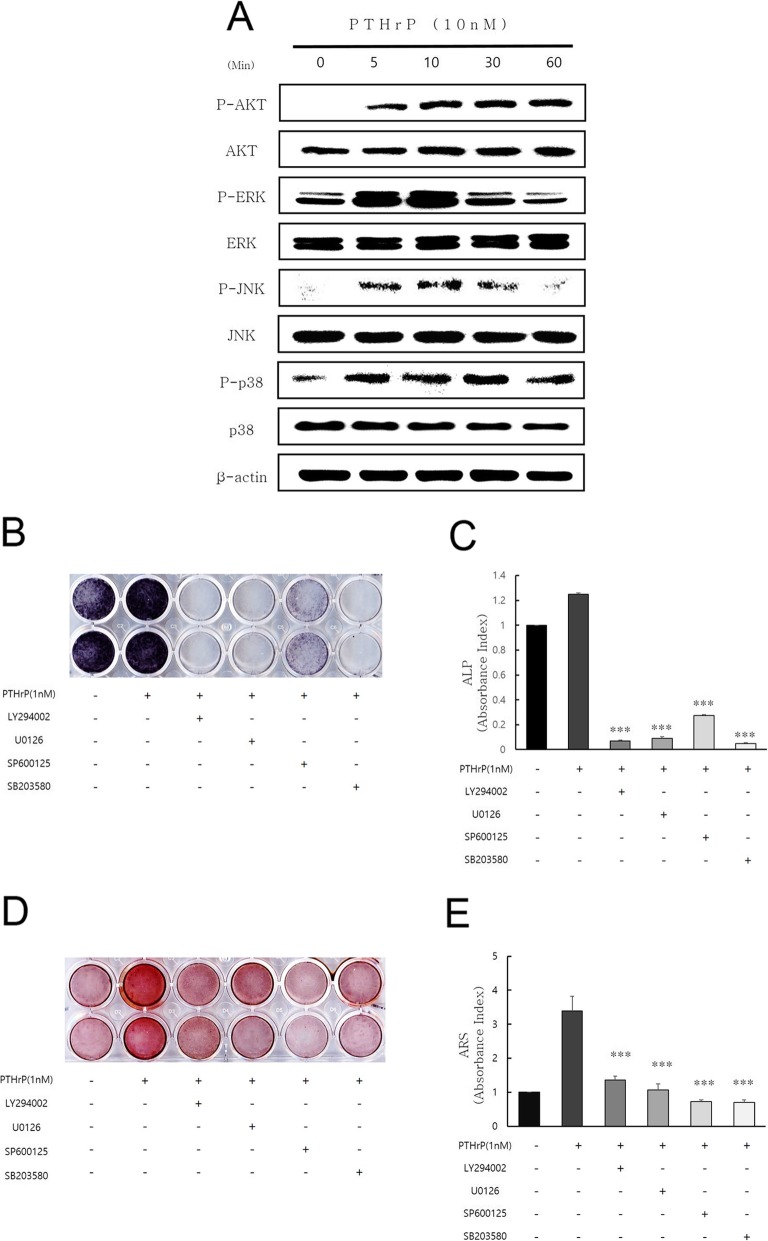
Effects of PTHrP on AKT, ERK, and JNK signaling pathways in hDPCs. (**a**) Effects of PTHrP on AKT, ERK, and JNK phosphorylation were assessed by Western blot analysis. Phosphorylation levels of AKT, ERK, and JNK were increased by PTHrP. (**b–e**) Effects of PTHrP and LY294002, U0126, and SP600125 on ALP staining and alizarin red S staining. The cells were pretreated with or without 10 μM LY294002, 10 μM U0126, or 10 μM SP600125 for one hour after treatment with 1 nM PTHrP. ALP staining (**b, c**) and alizarin red S staining (**d, e**) are shown. LY294002, U1026, and SP600125 significantly decreased PTHrP-induced ALP staining and mineralization (^***^P < 0.001 compared with the group treated with 1 nM PTHrP)

To investigate the signaling pathways involved in the PTHrP-induced odontogenic differentiation of hDPCs, the phosphorylation of AKT, ERK, JNK, and p38 in hDPCs was assessed by Western blot analysis. The results showed that treatment with 10 nM PTHrP increased the levels of p-AKT, p-ERK1/2, p-JNK, and p-p38 five minutes after treatment. These increases were sustained 30 min or 60 min after treatment (Fig. 3a).

To further investigate the role of AKT, ERK, JNK, and p38 signaling in the odontogenic differentiation induced by PTHrP, cells were pretreated with or without 10 μM LY294002 (AKT inhibitor; Cell Signaling Technology, #9901), 10 μM U0126 (ERK inhibitor; Cell Signaling Technology, #9903), 10 μM SP600125 (JNK inhibitor; Cell Signaling Technology, #8177), or 10 μM SB203580 (p38 inhibitor; Cell Signaling Technology, #5633) for one hour, followed by treatment with 1 nM PTHrP for seven or 14 days. LY294002, U0126, SP600125, and SB203580 also significantly (^***^P < 0.001) decreased the ALP staining and mineralized nodule formation induced by PTHrP (Fig. 3)b-e.

## Discussion

PTHrP is a paracrine regulator that plays an important role in bone growth and placental calcium transport [25]. It has physiological anabolic action in bone [26]. In vitro studies have suggested that PTHrP can modulate chondrocyte proliferation and differentiation partially by activating Gli3 [27]. PTHrP is also an important signal in osteoclast activation. It was shown to be essential in the process of tooth eruption in PTHrP-knockout mice rescued by a procollagen II-driven transgene. It plays an important role in cell proliferation and differentiation through PTH1R [23, 28].

PTHrP can affect proliferation and differentiation in osteoblasts. The PKC-dependent activation of the MAPK signaling pathway in rat mesenchymal progenitor cells was shown to stimulate osteogenic cell proliferation [29, 30]. PTHrP can increase the secretion of mineralization, ALP Activity, and osteocalcin, thereby activating peptides and protein and increasing cell proliferation and bone formation [10, 19]. The effect of PTHrP on odontogenic differentiation has not been reported. However, the odontogenic potential of osteostatin [fragment of PTHrP (107–111)] was demonstrated in a previous study [31]. Therefore, this study is the first to show the odontogenic potential of PTHrP in hDPCs. The reason for using hDPCs in this study was that most of the studies in systematic reviews reported positive results when hDPCs were used for hard tissue engineering. HDPCs have a demonstrated ability to proliferate even faster than bone marrow cells, to be homogenous, and possess an excellent capability to differentiate toward numerous cell lines [5, 7].

Cell viability was measured after treatment with PTHrP at concentrations of 1 nM, 10 nM, and 100 nM using the WST-1 assay. Previous studies have reported that when PTHrP was administered in excess, it affected dentin thickness and crevice formation [32]. Therefore, 1 nM and 10 nM of PTHrP were used in the present study. This result was consistent with the results of Ge X et al. [33].

The cell incubation times was determined based on a previous study [31]. The present study showed that different concentrations of PTHrP can induce odontogenic differentiation evidenced by the induction of ALP activity and the formation of mineralized nodules, as well as the upregulation of odontogenic markers. DMP1 and DSPP in odontoblasts are positive regulators of hard tissue mineralization with essential roles in the mineralization of dentin [34, 35]. Therefore, they were used as differentiation markers in the present study. Because there is a relationship between osteogenesis and odontogenesis [36], osteogenic differentiation markers, such as runt-related transcription factor 2 (Runx2), osterix (OSX), and osteocalcin (OCN) are also good markers for the effects of PTHrP on mineralization and have been studied in previous studies. PTHrP increased collagen type 1 (Col-1), osteopontin (OPN), OCN, and OSX mRNA and protein expression [10, 19]. As shown in Fig. 1, the relative mRNA levels of DSPP and DMP-1 were significantly higher in cells treated with PTHrP at 1 or 10 nM than those in cells not treated with PTHrP. The protein expression levels of DSPP and DMP-1 were increased in PTHrP-treated cells compared to the OM group without PTHrP treatment. The proteins were also significantly increased in the group treated with PTHrP at 10 nM after five days. In addition, as shown in Fig. 2, ALP activity and mineralized nodules were significantly increased in the PTHrP-treated group compared to the GM and no PTHrP groups. These results are consistent with the results of a study on the PTH effects on the osteo/odontogenic differentiation of DPCs [33]. These results suggest that PTHrP may have clinical benefit in pulp-dentin complex regeneration.

MAPK cascades have been shown to play an important role in the transduction of extracellular signals to cellular responses, including cellular proliferation and differentiation in mammalian cells. It has been reported that PTHrP/PTH treatment in proliferating osteoblasts can promote cell growth and that MAPKs play an important role in the effect of PTH and PTHrP on osteoblasts [30, 37, 38].

Tang et al. [39] reported that AKT and JNK were activated when apelin stimulated the proliferation of mouse osteoblastic MC3T3-E1 cells and that cell proliferation was inhibited by inhibitors of AKT and JNK. Another study reported that Runx2 increased P13K-AKT protein levels through the positive feedback loop of Runx2 and P13K-AKT. P13K-AKT signaling plays an important role in DNA binding and transcriptional activation of Runx2. Thus, osteoblast and chondrocyte differentiation and migration are connected. The role of AKT signaling in cell proliferation and differentiation has also been confirmed [40]. However, reports on PTHrP-related AKT signaling are insufficient.

This study investigated whether MAPK signaling and AKT signaling could affect PTHrP-mediated odontogenic differentiation. The inhibitor itself could have an endogenous effect to attenuate osteogenic differentiation. However, because the aim of this study was to investigate the underlying signaling mechanisms of PTHrP-mediated odontogenic differentiation, no inhibitor-alone group was included in the experimental conditions, based on previous studies [31, 41, 42]. The doses selected in the chemical inhibitor experiments were determined based on a previous study [41]. As shown in Fig. 3, the results showed that PTHrP increased the phosphorylation levels of AKT, ERK, JNK, and p38. The AKT inhibitor LY294002, ERK inhibitor U0126, JNK inhibitor SP600125, and p38 inhibitor SB203580 all inhibited the increase in ALP staining and mineralized nodule formation induced by PTHrP. These results suggest that PTHrP may be involved in odontogenic differentiation through the AKT, ERK, JNK, and p38 signaling pathways. However, further studies are needed to confirm the precise signaling mechanism involved in the PTHrP-induced odontogenic differentiation of hDPCs.

## Conclusion

In conclusion, this study demonstrated that PTHrP could promote odontogenic differentiation and mineralization in hDPCs by activating the AKT, ERK, JNK, and p38 signaling pathways. Therefore, PTHrP might be clinically useful for inducing dentin formation.

## Supplementary information

**Additional file 1.**

**Additional file 2.**

## Data Availability

The datasets generated or analyzed during the current study are available from the corresponding author upon reasonable request.

## Abbreviations

PTHrP: Parathyroid hormone-related protein;
ALP: Alkaline phosphatase
DMP: Dentin matrix protein
DSPP: Dentin sialophosphoprotein
ERK: Extracellular signal-regulated kinase
FBS: Fetal bovine serum
GM: Growth media
hDPC: Human dental pulp cell
HRP: Horseradish peroxidase
IRB: Institutional review board
JNK: C-Jun N-terminal kinase
MAPK: Mitogen-activated protein kinases
MEM: Minimum essential medium
OM: Odontogenic induction media
PBS: Phosphate-buffered saline
WST: Water-soluble tetrazolium
