# Supplementary figures and images for: Effect of parathyroid hormone-related protein on odontogenic differentiation in human dental pulp cells
Source: BMC Oral Health. 2020 Apr 10;20:101. doi: 10.1186/s12903-020-01085-8 (PMC7146980; doi:10.1186/s12903-020-01085-8)

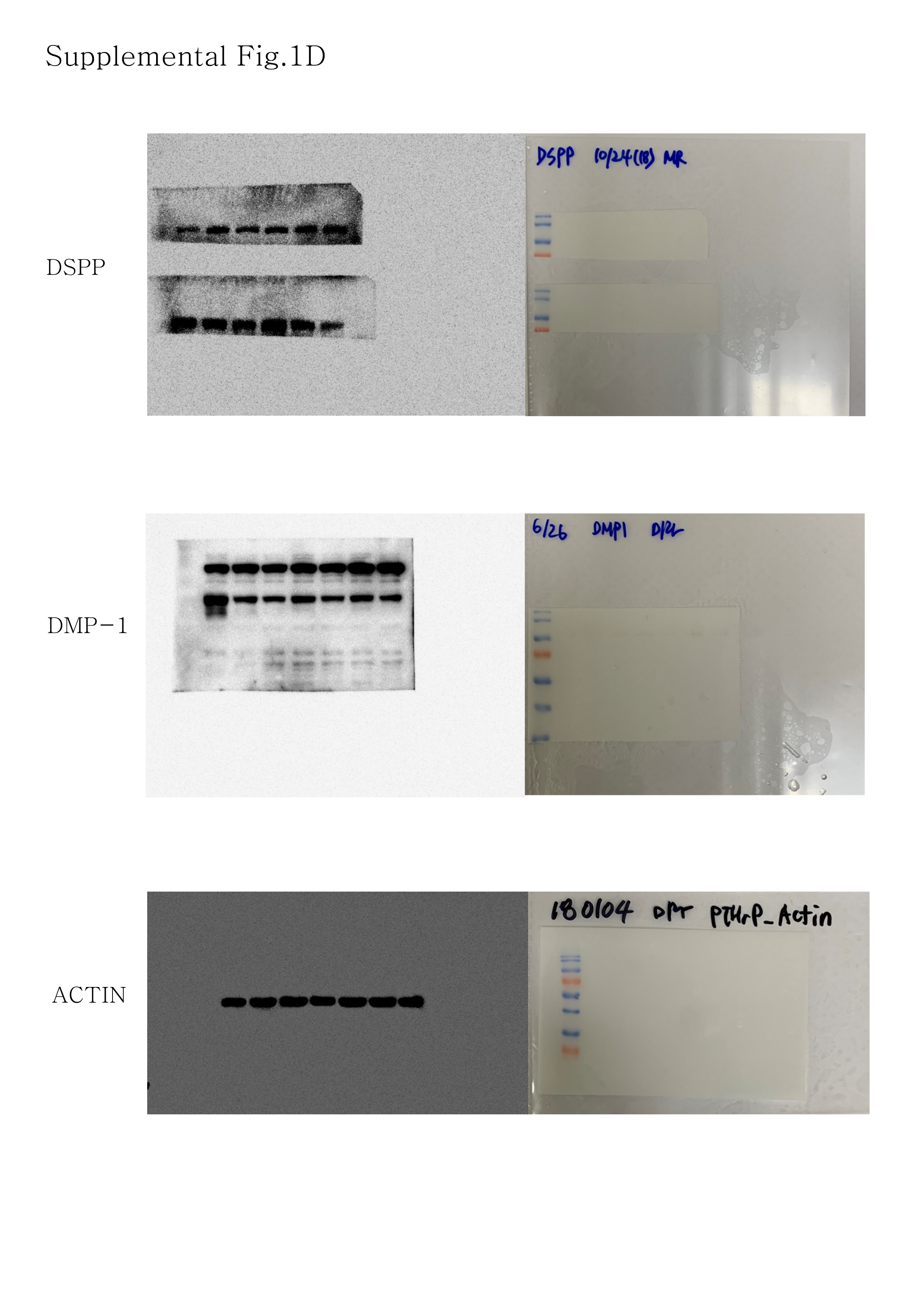

Supplement: Supplementary file 1 — Additional file 1. [file 12903_2020_1085_MOESM1_ESM.tif]

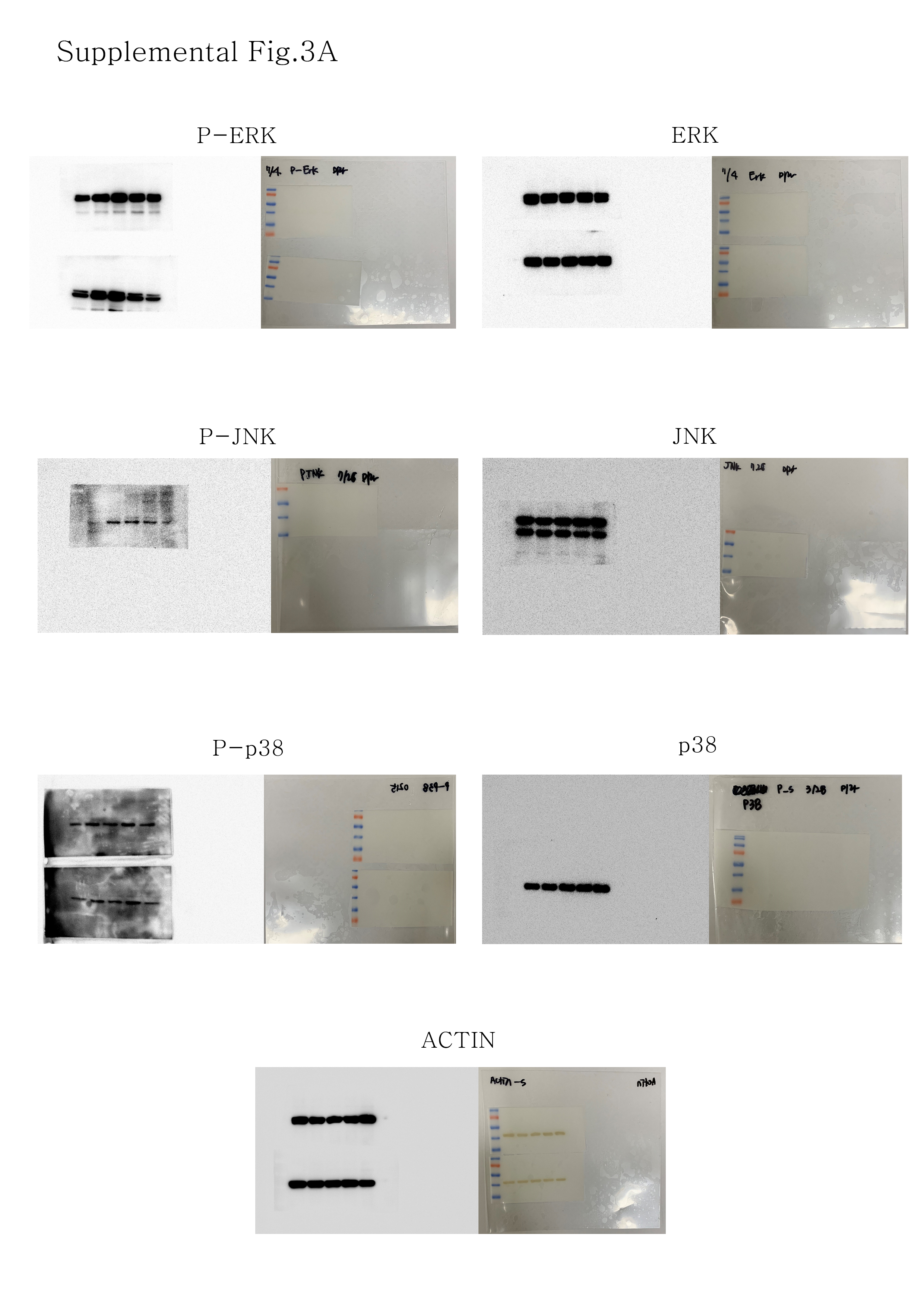

Supplement: Supplementary file 2 — Additional file 2. [file 12903_2020_1085_MOESM2_ESM.tif]
